# Supplementary material for: Vegetables contamination by heavy metals and associated health risk to the population in Koka area of central Ethiopia
Source: PLoS One. 2021 Jul 12;16(7):e0254236. doi: 10.1371/journal.pone.0254236 (PMC8274883; doi:10.1371/journal.pone.0254236)
Supplement: S1 File — (PDF) [file pone.0254236.s001.pdf]

Vegetables contamination by heavy metals and associated health risk to the population in Koka area of central Ethiopia

Leta Danno Bayissa[1]* and Hailu Reta Gebeyehu[1,#a]

[1]Ambo University, College of Natural and Computational Sciences, Department of Chemistry

P. O. Box 19

[#a] Current Address: Ethiopian Institute of Agricultural Research, Addis Ababa, Ethiopia

*Corresponding author: Email: bayissa.leta@ambou.edu.et

Supporting Information

S1 Table. Method detection limits for vegetable and soil samples analysis.

Heavy metals	IDL (mg/L)	Vegetable samples		Soil Samples	
		MDL (mg/L)	LOQ (mg/L)	MDL (mg/L)	LOQ (mg/L)
As	0.0001	0.0011	0.084	0.0016	0.185
Pb	0.0004	0.0007	0.057	0.0006	0.034
Cd	0.0003	0.0005	0.063	0.0008	0.035
Zn	0.0004	0.0007	0.084	0.0012	0.746
Cu	0.0006	0.0013	0.047	0.0015	0.043
Fe	0.0001	0.0004	0.036	0.0008	0.066
Mn	0.0001	0.0002	0.069	0.0004	0.070
Cr	0.0007	0.0014	0.033	0.0016	0.032
Hg	0.0006	0.0015	0.023	0.0017	0.021
Ni	0.0004	0.0011	0.018	0.0015	0.016
Co	0.0002	0.0013	0.024	0.0019	0.011

S2 Table. Percentage recovery values of the method used for soil sample digestion (Mean ± SD, n = 3)

Heavy Metal	Concentration before spiking (M± SDs) (mg/kg)	Amount spiked (mg/kg)	Concentration after spiking (M± SD) (mg/kg)	% Recovery	% RSD
Cr	0.414±0.01	5	6.04±0.014	112.52	2.42
Cd	0.06±0.012	5	5.86±0.013	116.00	20.0
Zn	1.069±0.035	5	6.55±0.015	109.62	3.27
Fe	0.453±0.053	5	6.04±0.023	111.74	11.69
Pb	0.430±0.021	5	5.93±0.227	110.00	4.88
Cu	0.263±0.001	5	5.76±0.011	109.94	0.38
As	0.324 ±0.059	5	5.92±0.233	111.92	8.21
Mn	0.177±0.005	5	5.65±0.006	109.46	8.48
Hg	0.165±0.008	5	5.26±0.017	101.9	4.84
Ni	0.35±0.008	5	5.47±0.014	109.4	2.28
Co	0.136±0.027	5	5.76±0.014	112.08	9.85

S3 Table. Percentage recovery values of the method used for tomato sample digestion (Mean ± SD, n = 3)

Heavy Metal	Concentration before spiking (M± SD) (ppm)	Amount spiked (ppm)	Concentration after spiking (M± SD) (ppm)	% Recovery	% RSD
Cr	0.12 ± 0.013	5	5.78±0.018	113.20	10.83
Cd	0.124±0.004	5	5.85±0.026	114.52	3.22
Zn	0.222±0.007	5	5.87±0.02	112.96	3.15
Fe	0.432±0.002	5	6.23±0.022	115.96	0.46
Pb	0.024±0.004	5	5.68±0.151	113.12	6.66
Cu	0.134±0.006	5	5.54±0.015	108.12	4.48
As	0.008± 0.001	5	5.8±0.132	115.84	8.50
Mn	0.277±0.004	5	6.15±0.004	117.46	1.44
Hg	0.032±0.001	5	5.33±0.001	105.96	3.12
Ni	0.009±0.001	5	5.28±0.021	105.42	11.11
Co	0.004±0.0001	5	5.23±0.016	104.52	2.50

S4 Table. Percentage recovery values of the method used for cabbage sample digestion (Mean ± SD, n = 3)

Heavy Metal	Concentration before spiking (M± SD) (ppm)	Amount spiked (ppm)	Concentration after spiking (M± SD) (ppm)	% Recovery	% RSD
Cr	0.390±0.03	5	5.10±0.02	94.20	7.69
Cd	0.230±0.004	5	5.40±0.01	103.40	1.74
Zn	0.354±0.01	5	5.73±0.02	107.52	2.82
Fe	0.330±0.06	5	5.03±0.17	94.00	8.18
Pb	0.262± 0.05	5	5.45±0.21	103.76	9.08
Cu	0.072±0.01	5	5.07±0.02	99.96	10.88
As	0.059±0.01	5	5.36±0.20	106.02	6.95
Mn	0.076±0.01	5	5.18±0.02	102.08	10.16
Hg	0.042±0.003	5	5.21±0.01	103.36	7.14
Ni	0.027±0.004	5	5.4±0.019	107.46	9.81
Co	0.0136±0.001	5	5.56±0.019	110.94	7.35

S5 Table. Optimal condition for soil and vegetables samples digestion procedures.

Trials	Reagent volumes (mL)			Temperature (°C)	Pressure (w)	Time (min)	Result /Observation
	HNO_3	HCl	Total				
1	4	8	12	70-130	25-55	20	Deep yellow
2	5	7	12	80-140	30-60	25	Deep yellow
3	6	6	12	90-150	35-65	30	Deep yellow
4	7	5	12	100-160	40-70	35	clear and pale
5	8	4	12	110-170	45-75	40	Clear and pale
6	***9**	***3**	**12**	***120-180**	***50-80**	***45**	***Clear and Colorless**
7	10	2	12	130-190	55-85	50	Clear with suspension
8	11	1	12	140-200	60-90	55	Clear with suspension

* indicates the selected optimal condition for soil and vegetables samples digestion procedure.

S6 Table. Parameters and variables used in the calculations of EDI, THQ and TCR

Parameters		Vegetable Types		References
		Tomato	Cabbage	
E_f (days)		365	365	-
E_D (years)		65	65	[1]
F_{IR} (g/day)		240	240	[2]
C_M (mg/kg dry weight)		Table 2	Table 2	This study
C_f		0.085	0.085	[3–5]
B_W (kg)		70	70	[1]
T_A (days)		23725	23725	-
Oral reference dose *(RfD)* *(mg/kg/day)*	As	0.0003	0.0003	[6]
	Pb	0.0035	0.0035	[7]
	Cd	0.001	0.001	[6]
	Zn	0.3	0.3	[8]
	Cu	0.04	0.04	[8]
	Fe	0.7	0.7	[8]
	Mn	0.14	0.14	[8]
	Cr	0.003	0.003	[7]
	Hg	0.0003	0.0003	[7]
	Ni	0.02	0.02	[8]
	Co	0.0003	0.0003	[8]
Oral cancer slope factor *(CPS_o)* *$(mg/kg/day)^{-1}$*	As	1.5	1.5	[6]
	Pb	0.0085	0.0085	[9]
	Cd	0.38	0.38	[10]
	Cr	0.5	0.5	[11]
	Ni	1.7	1.7	[8]

References

1. Woldetsadik D, Drechsel P, Keraita B, Itanna F, Gebrekidan H. Heavy metal accumulation and health risk assessment in wastewater-irrigated urban vegetable farming sites of Addis Ababa, Ethiopia. Int J Food Contam. 2017;4: 9. doi:10.1186/s40550-017-0053-y

2. WHO. The world health report 2002 : reducing risks, promoting healthy life. World Health Organization; 2002. Available: https://apps.who.int/iris/handle/10665/42510

3. Harmanescu M, Alda L, Bordean D, Gogoasa I, Gergen I. Heavy metals health risk assessment for population via consumption of vegetables grown in old mining area; a case study: Banat County, Romania. Chem Cent J. 2011;5: 64. doi:10.1186/1752-153X-5-64

4. Rattan RK, Datta SP, Chhonkar PK, Suribabu K, Singh AK. Long-term impact of irrigation with sewage effluents on heavy metal content in soils, crops and groundwater—a case study. Agric Ecosyst Environ. 2005;109: 310–322. doi:10.1016/J.AGEE.2005.02.025

5. Arora M, Kiran B, Rani S, Rani A, Kaur B, Mittal N. Heavy metal accumulation in vegetables irrigated with water from different sources. Food Chem. 2008;111: 811–815. doi:10.1016/j.foodchem.2008.04.049

6. Antoine JMR, Fung LAH, Grant CN. Assessment of the potential health risks associated with the aluminium, arsenic, cadmium and lead content in selected fruits and vegetables grown in Jamaica. Toxicol Reports. 2017;4: 181–187. doi:10.1016/j.toxrep.2017.03.006

7. Chang CY, Yu HY, Chen JJ, Li FB, Zhang HH, Liu CP. Accumulation of heavy metals in leaf vegetables from agricultural soils and associated potential health risks in the Pearl River Delta, South China. Environ Monit Assess. 2014;186: 1547–1560. doi:10.1007/s10661-013-3472-0

8. Javed M, Usmani N. Accumulation of heavy metals and human health risk assessment via the consumption of freshwater fish Mastacembelus armatus inhabiting, thermal power plant effluent loaded canal. Springerplus. 2016;5: 776. doi:10.1186/s40064-016-2471-3

9. Kamunda C, Mathuthu M, Madhuku M. Health Risk Assessment of Heavy Metals in Soils from Witwatersrand Gold Mining Basin, South Africa. Int J Environ Res Public Health. 2016;13. doi:10.3390/ijerph13070663

10. Yang J, Ma S, Zhou J, Song Y, Li F. Heavy metal contamination in soils and vegetables and health risk assessment of inhabitants in Daye, China. J Int Med Res. 2018;46: 3374–3387. doi:10.1177/0300060518758585

11. Zeng F, Wei W, Li M, Huang R, Yang F, Duan Y. Heavy Metal Contamination in Rice-Producing Soils of Hunan Province, China and Potential Health Risks. Int J Environ Res Public Health. 2015;12: 15584–93. doi:10.3390/ijerph121215005
