# Supplementary material for: Vegetables contamination by heavy metals and associated health risk to the population in Koka area of central Ethiopia
Source: PLoS One. 2021 Jul 12;16(7):e0254236. doi: 10.1371/journal.pone.0254236 (PMC8274883; doi:10.1371/journal.pone.0254236)
Supplement: Attachment — Submitted filename: Reviewers comments.docx [file pone.0254236.s003.docx]

Review Comments for PONE-D-21-05216, [EMID:a62f1aa366bb5f78]

I (Dr. Arif Husain, Department of the Soil and Environmental Science, Ghazi University, Dera Ghazi Khan, Pakistan) reviewed the Manuscript PONE-D-21-05216. Authors have worked hard in formulating the current manuscript. With the following’s amendments, the Manuscript may be considered for publication in the Journal “PLOS ONE”.

1- In Abstract, TCR write completely when write first time and can be abbreviated next time writing.
2- WHO criteria for critical limits (maximum, minimum and threshold) for the heavy metals in cabbage and tomato were not considered. Why?
3- What are the concentrations of the heavy metals in the lake KOKA water due to industries existing near the KOKA lake? Heavy metals concentrations in the KOKA Lake water were not included in the Manuscript. Why?
4- In methodology, total of 2 cabbage and two tomato samples from both field areas were collected. Were these samples enough for manuscript data?
5- In methodology; Page No.4 and Line number 80; convert “steeliness” into “stainless”.
6- Total 4 soil samples from the study site were taken. Only 4 samples are enough for the manuscript data?
7- In “Result and Discussion” Table No. 1. No need to write word “Beneath”. It is better to write “area under tomato cultivation”. Rest of the correction should be incorporated.
8- Page No. 9. Line No. 186; values 2.06 to 2.30 for %MC are not according to the Table No. 1. The values for %MC are 24.53 and 19.65. Formerly mentioned values (2.06 & 2.30) are for %OM according to Table No. 1. Similarly, in the Line No. 186 and 187; values 1.09 and 1.41 are for %OC, not for %OM, so, correct them according to the Table No. 1.
9- In the line No. 190; value for %OC 44.9% seems to be doubted. So, author should check it.
10- Mention effects of heavy metals under discussion on human body like causing different diseases and disorders in the human beings.
11- Page No. 13, Line No. 281; value 16.96 is not according to its concerned Table No.3.
12- Line No. 282; value for Ni is 09, which is not according to the Table No. 3.
13- English language for the manuscript should be improved.

Thanks

Best Regards

Dr. Arif Husain

Department of Soil and Environmental Sciences

Faculty of Agricultural Sciences

Ghazi University, Dera Ghazi Khan, Pakistan.
