# Supplementary material for: Vegetables contamination by heavy metals and associated health risk to the population in Koka area of central Ethiopia
Source: PLoS One. 2021 Jul 12;16(7):e0254236. doi: 10.1371/journal.pone.0254236 (PMC8274883; doi:10.1371/journal.pone.0254236)
Supplement: Attachment — Submitted filename: Response to Reviewers.docx [file pone.0254236.s004.docx]

**General Remark**

Above all, we are very much grateful for the commitment of PLOS ONE Editors and staffs. We really appreciate the swift response and genuine & very constructive review comments we have received on our manuscript. We have gone through our manuscript once again and tried to address all the comments forwarded as much as possible.

**Response on the points raised by the Academic Editor**

1. I have carefully looked at the abbreviations we have used and defined when it was first mentioned.
2. WHO/FAO based critical limits/recommended values for heavy metals in both soil and vegetable have already been included (cf. Tables 2&3).
3. We have recently reported levels of heavy metals in both soil and vegetables from the nearby areas and stated that this project is the continuation of that. Elevated heavy metals were reported (cited in this manuscript, <https://doi.org/10.1371/journal.pone.0227883>) as these areas are home for many small-scale industries.
4. We have checked once again the journal format and made corrections accordingly.
5. We have gone through our manuscript and tried our best to make necessary corrections as per the suggestion.

**Response to Reviewers Comment**

**Reviewer #1**

The comments forwarded are very constructive and helped us to enrich our paper.

i) Corrections were made as per the suggestion
ii) We have corrected the comment
iii) We have corrected it.

We have gone through our manuscript once again and tried to correct some grammatical mistakes and type errors.

**Reviewer #2**

We really appreciate the reviewer for the genuine and constructive comments. The comment given are very helpful and therefore, all the suggestions have been given due attention and corrections have been made accordingly.

1. Correction was made
2. WHO/FAO based critical limits/recommended values for heavy metals in both soil and vegetable have already been included (cf. Tables 2&3).
3. Our focus in this study was to evaluate the levels of heavy metals in the soil and commonly used vegetables and we haven’t considered the investigation of heavy metals in Koka water this time.
4. For each vegetable from each filed (as explained under methodology section 2.2), five different samples were collected and pooled together to obtain a composite sample. That means five cabbage sample from Koka Ejeresa comprising one representative sample after mix and Five similar samples from Koka Negewo area. The same is true for the cabbage samples as well.
5. Line 80 >> corrected accordingly.
6. Soil samples were taken from the exact location where vegetables have been samples. So, five different samples were taken and pooled together to obtain representative sample from each location.
7. Table 1 >> corrected accordingly
8. That was a mistake and it has been corrected, thank you very much.
9. I agree that the value seems doubted, but we have checked the article once again and it is what has been reported.
10. We have stated the effects of heavy metals on human being under introduction section. As there are ample of scientific articles discussing the same, we have made abstinence on going in detail into it.
11. Corrected
12. Corrected
13. We have gone through our manuscript and made necessary corrections as per the suggestions.
