# Supplementary material for: Vegetables contamination by heavy metals and associated health risk to the population in Koka area of central Ethiopia
Source: PLoS One. 2021 Jul 12;16(7):e0254236. doi: 10.1371/journal.pone.0254236 (PMC8274883; doi:10.1371/journal.pone.0254236)
Supplement: Attachment — Submitted filename: reviewer comments PONE-D-21-05216.docx [file pone.0254236.s002.docx]

Review Comments:

The article titled “Vegetables contamination by heavy metals and associated health risk to the

population in Koka area of central Ethiopia.” Is worth accepting, as this study is well designed and is comprehensive, covering all the related elements. But there are few things which I suggest are as follows:

Few suggestions should be incorporated as under:

i) Clearly defined the abbreviations used in the manuscript
ii) Line no. 8 correct the word “glob”
iii) Line no. 373 “Figure 1 here”. It will be replaced with the Figure 3 given below on line no. 375.

Over all I have seen some grammatical mistakes and few typing mistakes in the manuscript. As they are treated as accepted.
